# Deterministically selected rare taxa drive changes in community composition in drinking water biological activated carbon filters

**DOI:** 10.1186/s40793-025-00820-4

**Published:** 2025-11-24

**Authors:** Dominic Quinn, Marta Vignola, Jeanine Lenselink, Graeme Moore, Stephanie Connelly, Caroline Gauchotte-Lindsay, Umer Ijaz, William T. Sloan, Cindy J. Smith

**Affiliations:** 1 James Watt School of Engineering, University of Glasgow, Glasgow, G12 8QQ UK; 2 Scottish Water, 6 Castle Drive, Dunfermline, KY11 8GG UK

**Keywords:** Biological activated carbon, Biofiltration, Microbial community assembly, 16S rRNA, Drinking water treatment

## Abstract

**Background:**

Biofiltration offers a sustainable, low-energy solution for drinking water treatment but suffers from inconsistent performance due to complex microbial dynamics. Current studies lack insight into early biofilter microbial community assembly. Here we perform a high-resolution spatial and temporal investigation of biomass accumulation and community development within biological activated carbon (BAC) filters over the first 6 months of operation.

**Results:**

We found that initial biomass accumulation is not linear, instead characterised by periods of growth and decay. Mass balance identified an estimated + 6.54 × 10^8^ new cells daily during the growth phase (days 34–62), falling to a loss of 1.69 × 10^9^ by the decay phase (days 83–162). There was no significant increase in richness until the decay phase (ANOVA *p* values > 0.05 between days 34, 62 and 83). Significant stratification (ANOVA *p* values < 0.05) was observed with bed depth with 79% (± SD2.7%) of biomass found in the top 15 cm of the filter bed, the bottom section (90 cm) had 36.5-fold lower biomass. An abundant community of 20 primary colonisers made up to between 20 ± SD8% and 80 ± SD5% of the total community and persisted over time. This community increased in absolute number during the growth phase (140% increase) however remained stable after this. Conversely rare taxa were found to continue to increase into the decay phase (131% between days 62–162). Core community analysis and neutral modelling of the seeding influent water and the biofilter found that the abundant taxa stochastically assembled early from the water, while the rare taxa driving changes in diversity, were selected by deterministic factors within the filter bed with 38% advantaged by the filter environment, compared to only 20% of the persistent abundant community.

**Conclusion:**

This study demonstrates that biofilter microbial communities undergo dynamic changes, with abundant early colonizers persisting steadily while rare taxa drive fluctuations in biomass through phases of growth and decay. Understanding these microbial dynamics and ecological interactions can inform engineering strategies to optimize biofilter performance, enhancing water treatment efficiency by targeting key microbial groups throughout filter maturation.

**Supplementary Information:**

The online version contains supplementary material available at 10.1186/s40793-025-00820-4.

## Background

Sustainability in drinking water treatment has become a global issue, leading to low cost, low carbon solutions being highly sought after. An established technology with considerable potential is biofiltration, encompassing systems such as slow sand filters and biological activated carbon (BAC) filters. Biofiltration offers multiple concurrent benefits for water treatment, as the biofilter microbiome facilitates the removal of nutrients [34], emerging contaminants [38] and pathogens [47]. Since the main mechanisms of treatment is through biological activity or passive adsorption, little additional energy is required for operation.

However, biofiltration is not without its challenges, and a drawback is often reliability. The diverse nature of biofilms associated with biofiltration make biological function difficult to predict, direct, and control [25, 27], potentially leading to performance issues. Ultimately, the reliability of the process and the quality of water produced by biofiltration require improvement. This can be achieved through a more comprehensive understanding of the biology underpinning filter bed performance. [21, 27, 48, 49].

While studies have examined biomass accumulation and microbial community development within biofilters [1, 13, 21–23, 27, 49, 58], documenting increases in biomass over time [3, 22, 23, 30, 49] and shifts in community composition with depth [4, 10, 22, 23, 25, 31, 51], most have focused on mature, full-scale filters or on community changes driven by seasonality or pre-treatment effect. Previously it has been shown that the assembly of biofilter communities are influenced by a range of factors, including temperature, media type and the initial community of the influent water [5, 15, 22, 23, 52]. This suggests that while the biological community may ultimately differ between locations and source water [5], selective interventions could potentially be deployed to drive the community in a desired direction. However, despite the recognised influence of source water on biofilter communities, few studies have examined the initial stages of community assembly during start-up or the fine-scale temporal and spatial succession that occurs as the biofilter matures. This understanding is urgently needed if we are to direct and predict community assembly to enhance the effectiveness of biofilters and the predictive capability of any interventions.

A further limitation of current research is that microbial community dynamics are often described solely in terms of relative abundance [10, 25, 51] which does not reflect changes in absolute cell numbers. This issue is particularly relevant in biofilter environments, where total biomass varies over time and with depth. Although relative abundance data provide useful comparative insights, they fail to account for fluctuations in overall biomass, thereby obscuring patterns in the absolute abundance of individual taxa. Consequently, a clearer understanding of microbial community development in absolute terms of biomass is required. To address this knowledge gap, we applied 16S rRNA amplicon sequencing and quantitative assessments of microbial community dynamics to characterise biofilter community assembly at high temporal and spatial resolution during 6 months of surface water treatment. Specifically, we investigated how microbial cells within the biofilter accumulate over time and sought to identify the taxa responsible for shifts in community diversity. We explore if a core microbiome is present from start-up and if this is inoculated from the influent water. Furthermore, we investigate the ecological processes by which organisms are selected by the biofilter. We show that overall biomass increased during operation but followed alternating phases of growth and decline. A stable core of abundant primary colonisers persisted, yet their absolute numbers remained unchanged over time. Instead changes in biomass and diversity were driven by the gain and loss of rare taxa, largely influenced by deterministic selection.

## Materials and methods

### Biofilter set-up and operation

**Fig. 1:**
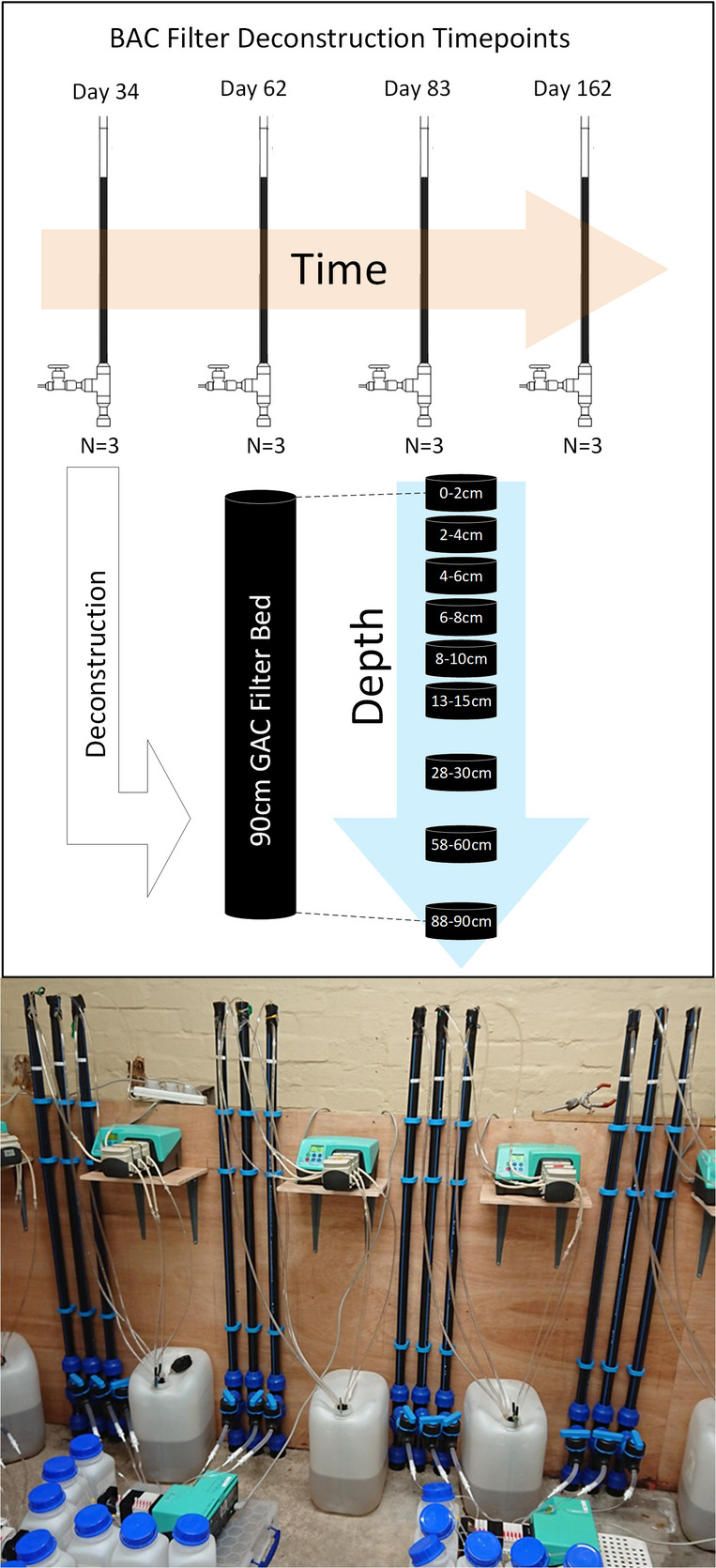
Schematic of spatial and temporal media sampling during the experiment run and photograph of the operational biofilter system in-situ

A set of twelve laboratory-scale filters, each containing a 90 cm GAC bed and constructed from PE80 pipe, was developed to replicate the microbial community dynamics of full-scale BAC filters from start-up through 6 months (162 days) of operation. The BAC filters were fed pre-filtered (10 µm retention) (Spectrum®, UK) raw surface water collected from Scottish Water’s Patsehill Reservoir. Details of the operating parameters and GAC characteristics can be found in Table 1. Details of biofilter construction can be found in S1 and Fig. S1. Details of biofilter deconstruction and sampling of GAC, influent and effluent water can be found in S2 (Fig. 1).

**Table 1:** Operating parameters, influent and GAC attributes of the biofilters used in this study

Influent water	Surface water (prefiltered 10 µm)
Influent pH	7.9 ± 0.16
Influent conductivity	117.5 ± 4.7
Influent temperature	21–23 °C
Bed length	90cm
Bed diameter	2.6 cm
Bed area	5.32 cm^3^
Flow rate	63.7 ml/h
Volume	477.9 cm^3^
HLR	0.12 m/h
EBCT	7.5 h
GAC (Cabot Norrit GAC 1240 W, Cabot Corporation, Boston, MA, USA)	
Packed bed density (WW)	4.5 ± 0.1 kg/cm^3^
Dry weight in column (APPROX)	230 g
Uniformity coefficient	1.7
Particle size > 1.7 mm	Min 10 mass%
Particle size < 0.425	Max 5 mass%

### ATP analysis

BacTiter-Glo™ ATP reagent mix (Promega, Madison, WI, USA) was prepared following the manufacturer’s instructions, and 350 µl was aliquoted in sterile Eppendorf tubes. Separate sterile Eppendorf tubes were prepared in duplicate with the addition of 200 mg of GAC (wet weight) and 100 µl of phosphate buffer. The ATP reagent and GAC in phosphate buffer were incubated for a minimum of 3 min at 30 °C. The GAC/phosphate buffer was vortexed before 300 µl of ATP reagent was added. The sample was then vortexed for 5 s, incubated in the dark for 1.5 min, and vortexed every 30 s [50]. After incubation, 200 µl of supernatant was transferred into a fresh sterile Eppendorf tube and measured immediately with a 10-s integration on a Promega GloMax® 20/20 Luminometer (Promega, Madison, WI, USA). Results were converted to ATP concentrations using a calibration curve established with dilutions of ATP (10mM, Promega, Madison, WI, USA) over a concentration range of 1 to 0.005μM of ATP added to 200mgWW of GAC previously deactivated by washing with phosphate buffer and incubating in a 60 °C water bath for at least 21 h. A conversion factor of 8.9 × 10^−17^ gATP/cell [8] was used to convert ATP concentrations into cell abundances.

### Mass balance analysis

For the biofilter, at each deconstruction time-point, ${(t}_{i})$, the total cell count (Cells/mL), as measured by flow cytometry, was multiplied by the flow rate ($Q$=1 mL/min) and the elapsed time to the next time point $({t}_{i+1}-{t}_{i})$. It was assumed that the cell concentration remained constant between two consecutive time points. This approach enabled the estimation of the total number of cells entering and exiting the system with the influent and effluent water, respectively, using the average Total Cell Count of the influent water samples (n = 4) and the averages of the effluent water samples from biofilters (n = 3). The total number of cells present in the filter at each deconstruction point was estimated by multiplying the average cell measurements obtained through ATP analysis at different depths (${q}_{f}^{GAC}=$ average from the biofilter triplicates) by the wet weight of the Granular Activated Carbon (GAC) at each bed depth $\left({M}_{f}^{GAC} \right)$ measured at the time of deconstruction. Applying a mass balance analyses the number of cells lost (due to decay) or acquired (due to growth) ${\Delta }_{cells}^{take down}$ was estimated following Eq. 1. Subsequently, the daily transformation rate (due to growth or decay) of cells was estimated following Eq. 2. Positive numbers of the ${\Delta }_{cells}^{take down}$ would indicate an overall growth of the biomass within the filter, while negative numbers would indicate an overall decay.

1 $$\begin{aligned} & \sum\limits_{{i = 0}}^{{TakeDown}} {\left[ {(TCC_{{t_{i} }}^{{IN}} - TCC_{{t_{i} }}^{{EFF}} )*\left( {t_{{i + 1}} - t_{i} } \right)} \right]} *Q \\ & = \sum\limits_{{f = 0}}^{{90cm}} {M_{f}^{{GAC}} } *q_{f}^{{GAC}} + \Delta _{{cells}}^{{takedown}} \\ \end{aligned}$$

2 $${\text{Transformation}}\;{\text{Rate}} = \frac{{\Delta_{cells}^{take down} }}{{t_{take down + 1} - t_{take down} }}$$

### DNA extraction

DNA was extracted from GAC and influent water from both filter factions (1.2 µm, 0.22 µm) using the FastDNA spin kit for soil (MP Biomedicals, Irvine, CA, USA) according to the manufactures protocol. Extraction blanks, consisting of reagent-only controls, were carried through the entire DNA extraction and PCR workflow as negative controls. Samples of GAC were removed from -80°C and thawed on ice before 0.4g was aseptically transferred to Lysis Matrix E tubes supplied with the FastDNA spin kit. Sterivex™ (Merck KGaA, Darmstadt, Germany) filters were pried open and transferred to Lysis matrix E tubes aseptically. MF200 glass fibre membranes (Fisher brand, Loughborough, UK) were cut in two and each half was transferred to a separate Lysis matrix E tube. DNA from the glass fibre filters were combined after extraction. DNA was extracted following the protocol supplied with the FastDNA kit with an extended centrifuge time (15 min) at step 5. Final DNA was eluted in 50 µl DES water supplied with the kit. DNA was quantified on a Qubit 4 Fluorometer (Invitrogen, Waltham, Massachusetts, USA) using the Qubit™ dsDNA HS quantification assay Kits (Invitrogen, Waltham, Massachusetts, USA). DNA was also visualised on a 1% agarose gel (run at 90 V for 40 min) using SYBR Safe reagents (Invitrogen, Waltham, Massachusetts, USA). DNA was stored at − 80 °C until further processing.

### Quantitative PCR

16S rRNA gene quantification was carried out using primers 1369F (CGGTGAATACGTTCYCGG) and 1496R (GGWTACCTTGTTACGACTT) and *Taq*Man probe 1389 (CTTGTACACACCGCCCGTC) [46]. Primers and probe were synthesised by Eurofins Scientific (Eurofins Scientific, Luxembourg City, Luxembourg). BioRad iTaq universal supermix reagents (Bio‑Rad Laboratories, Hercules, California, USA) were used with 1 µl of template. Template DNA was diluted 1:50. Premade standards [43] were run at fivefold dilutions from 1.3 × 10^8^ to 1.6 × 10^3^ copies. Quantitative PCR was carried out on a Quantstudio 3.0 Real time PCR system (Applied Biosystems, Thermo Fisher Scientific, Waltham, Massachusetts, United States) for an initial hold stage of 95 °C for 10 min, followed by 40 cycles of 95 °C for 10 s and 60 °C for 30 s.

### 16S rRNA Illumina library preparation

End point PCR was carried out using primers F515 (GTGYCAGCMGCCGCGGTAA) and R926 (CCGYCAATTYMTTTRAGTTT) covering the V4/V5 region of the 16S rRNA gene. Primers were synthesised by Eurofins Scientific (Eurofins Scientific, Luxembourg City, Luxembourg). Each primer had an attached Illumina adapter sequence and the forward primer a varying 12bp Golay barcode for each sample [29, 35]. Template DNA was diluted to a concentration of 0.5 ng µl^−1^, and 2 µl of this template was added to each reaction. No-template controls contained 2 µl of ultrapure water. PCR reactions (25 µl) contained 0.6 µM of each primer, 2 mM MgCl_2_, and 0.2 mM total dNTPs (QIAGEN GmbH, Hilden, Germany), prepared using HotStarTaq reagents (QIAGEN GmbH, Hilden, Germany). Amplification was performed on an Applied Biosystems 2720 thermal cycler (Applied Biosystems, Thermo Fisher Scientific, Waltham, MA, USA). The PCR program consisted of an initial denaturation at 95 °C, followed by 25 cycles of 95 °C for 30 s, 55 °C for 30 s, and 72 °C for 30 s, with a final elongation step at 72 °C for 10 min. PCR products were cleaned using AMpure XP beads (Beckman Coulter Life Sciences, Indianapolis, IN, USA) following the recommended protocol with a 0.7:1 bead ratio. Cleaned products were then pooled equimolarly and sent to the Earlham Institute (Norwich, UK) for Illumina Miseq 300 × 300 bp sequencing. Sequences were deposited in the National Center for Biotechnology sequence read archive under project accession PRJNA1309034.

### Data analysis

Raw sequencing data was processed to generate amplicon sequence variants (ASVs) using the Qiime2 pipeline and DADA2 algorithm [2]. Reads were demultiplexed and visualised following this workflow, then quality-trimmed at 240 bp for the forward reads and 200 bp for the reverse reads. Truncation lengths were chosen based on per-base quality profiles, which showed a decline in sequence quality toward the 3′ end, particularly for the reverse reads. Forward and reverse reads were merged to generate ASVs and dereplicated to generate abundance data. Maftt [14] was used to align ASVs and FastTree [32] was used to generate the rooted phylogenetic tree. Taxonomy was assigned with the Bayesian lowest common ancestor algorithm against the Silva v138 database. Microbial community data were processed and analysed using the R studio (2023.09.1 Build 494) and the phyloseq [24], vegan [28], ggplot2 [56], and tidyverse packages [57]. OTU tables were imported from BIOM format and rarefied to the minimum sequencing depth across samples to normalize read counts. Taxonomic annotations were cleaned, and contaminants (e.g., Chloroplast, Mitochondria, and unassigned taxa) were removed. Sample metadata were filtered to retain specific experimental groups of interest, and samples were grouped by time point and depth. ASVs were renamed for brevity using a custom mapping file. To assess biofilter performance, statistical tests were carried out on biological parameters collected over time. *P* values were calculated by PERMANOVA, one and two-way ANOVA and Tukey HSD 95% confidence intervals. Absolute abundances were calculated by multiplying 16S rRNA gene relative abundances from sequence data by16S rRNA gene qPCR data [12]. Differential abundance analysis was performed using ANCOM-BC 2, with multiple testing correction (Holm method), structural zero detection, and a significance cutoff of FDR-adjusted *p* < 0.05. Taxa with absolute log fold change ≥ 2 were considered biologically relevant and visualized accordingly [20]. For the core microbiome analysis an occupancy-abundance framework was applied where the occupancy of each taxon (i.e., proportion of samples in which it was detected) was plotted against its mean relative abundance. To identify core taxa, ASVs were ranked based on a composite index combining detection frequency and replication consistency across grouped samples. Bray–Curtis similarity was iteratively calculated as ranked ASVs were added, and an “elbow point” was used to define the optimal core set. Additionally, Sloan's neutral community model (NCM) was fitted to test whether ASVs detection frequencies deviated from neutral expectations. Taxa that were significantly more or less frequent than predicted were categorized as “Above” or “Below” the NCM, respectively [40].

## Results

### Biomass growth in the filter bed characterised by periods of growth and decay

**Fig. 2:**
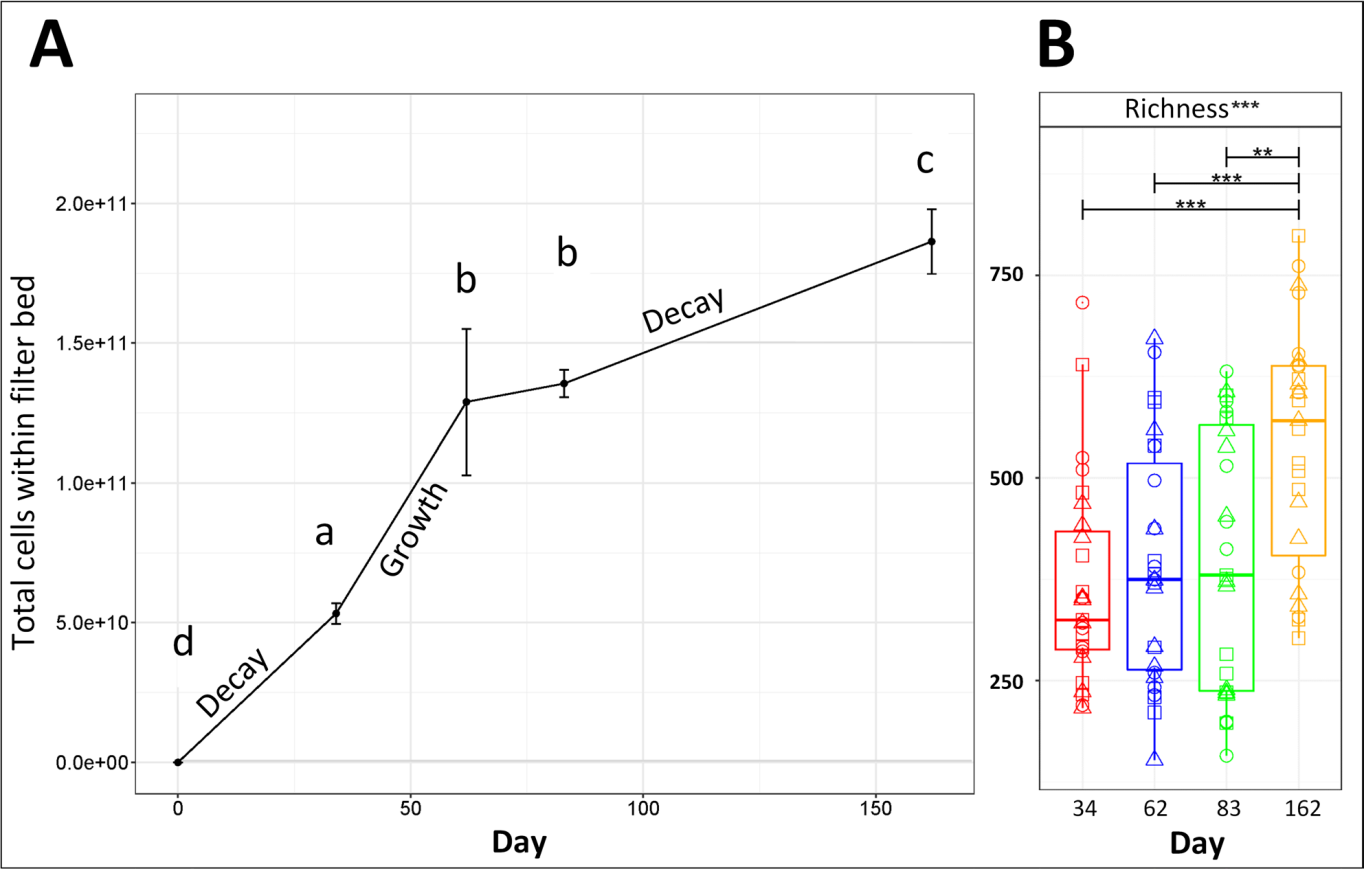
Estimated cells measured through ATP analysis throughout the entire filter bed over time with periods of growth or decay highlighted as calculated by mass balance. Flow cytometry was used to measure total cell counts in the influent and effluent water, thus quantifying the number of cells entering and exiting the biofilter system. ATP was measured from GAC at each depth, averaged on a per-cell basis, and used for the mass balance calculation. Further details can be found in Fig. S2. **A** Different letters (a–d) indicate statistical differences between different timepoints (days 34, 62, 83 and 162) (One-way ANOVA and Tukey HSD, *p* value < 0.05 for significance). **B** Species richness throughout the filter bed over time. Significance bars generated by one-way ANOVA

Microbial dynamics within the entire filter were followed for 162 days from start up, showing an increase in biomass and biological activity (Fig. 2) over time.

Biomass, based on ATP measurements, (Fig. 2A) found cells significantly increased over time, culminating in highest biomass on day 162 (1.8 × 10^11^ ± SD1.1 × 10^10^, one-way ANOVA and Tukey *p* value < 0.001). ATP measurements through the filter bed strongly correlated with qPCR data (Pearson’s correlation coefficient 0.97, *p* value 1.0 × 10–8) (Fig. S3). The steepest increase in cell number was observed between days 34 (5.3 × 10^10^ ± SD3.7 × 10^9^) and 62 (1.2 × 10^11^ ± SD2.6 × 10^10^). Mass balance calculations identified this timeframe as when the growth rate was at its highest, resulting in an estimated + 6.54 × 10^8^ new cells daily. Prior to this, despite the increase in cell numbers, was a period of net decay (day 0–34). Between days 62 and 162, growth rate appears to slow and was accompanied by cell decay, resulting in a net loss of an estimated − 1.23 × 10^9^ total cells/day between days 62 and 83 and − 1.69 × 10^9^ total cells/day between days 83 and 162.

Species richness significantly increased overall from day 34 to 162 (ANOVA *p* values ≤ 0.0017, 367.3 ± SD125.4 to 538.8 ± SD148.1). However, no significant differences in richness were observed between any of the earlier timepoints, including the growth period (Fig. 2B).

As biomass and richness in the entirety of the filter bed were found to increase over time through varying periods of growth and decay, we next explored how these periods related to time and depth changes in the abundance and composition of the microbial community.

**Fig. 3:**
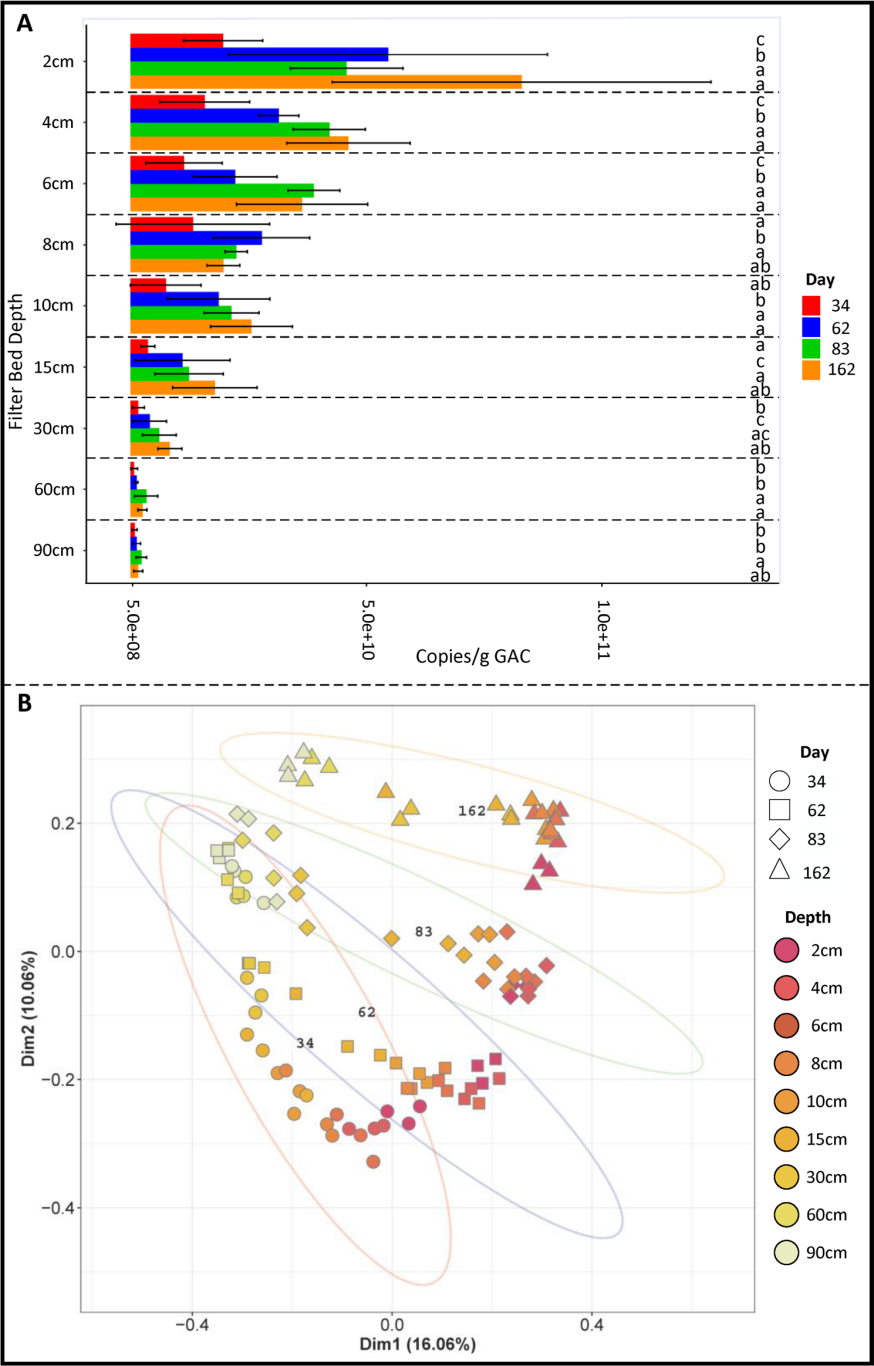
**A** 16S rRNA copies through the depth of the filter bed and over time. Letters indicate statistical similarity over time within each depth (one-way ANOVA and Tukey HSD). **B** PCoA of UniFrac distance through the depth of the filter bed and over time. Ellipses indicate the standard deviation of each sampling day from the centroid of the group (red—day 34, blue—day 62, green—day 83, orange—day 162)

The abundance of 16S rRNA genes generally decreased with sampling depth and increased over time (Fig. 3A). The vast majority, 79% (± SD2.7%) of 16S rRNA genes were found in the top 15 cm of the filter bed (Fig. 3A). Gene abundances were highest in the top 2 cm section, increasing 4.2-fold from day 34 to day 162 (2.0 × 10^10^ ± SD4.2 × 10^9^ to 8.4 × 10^10^ ± SD2.0 × 10^10^ 16S rRNA copies/g WW GAC). The 90 cm section of the filter bed contained 36.5-fold lower 16S rRNA genes than the top 2 cm but increased 2.64-fold from day 34 to 162 (8.7 × 10^8^ ± SD2.8 × 10^8^ to 2.3 × 10^9^ ± SD5.7 × 10^8^ 16S rRNA copies/g GAC), although this was not a statistically significant increase. Copy numbers were found to increase significantly over time and through depth in the top 15 cm (ANOVA and Tukey HSD *p* values < 0.05). In the bottom sections of the filter bed (60-90 cm) no significant change was detected with depth at any timepoint (ANOVA and Tukey HSD *p* values > 0.05).

Decreasing 16S rRNA gene abundances with depth and increasing values over time were accompanied by shifts in community composition, with communities in the bottom 30–90 cm becoming increasingly similar over time (Fig. 3B). PERMANOVA analysis identified sampling depth as explaining the majority of variance, 24% (*p* value ≤ 0.001), while time explained 15% of variance (*p* value ≤ 0.001). The interaction between time and sampling depth was also significant, explaining 25% of variance between communities.

### Changes in biomass and diversity are driven by rare taxa

**Fig. 4:**
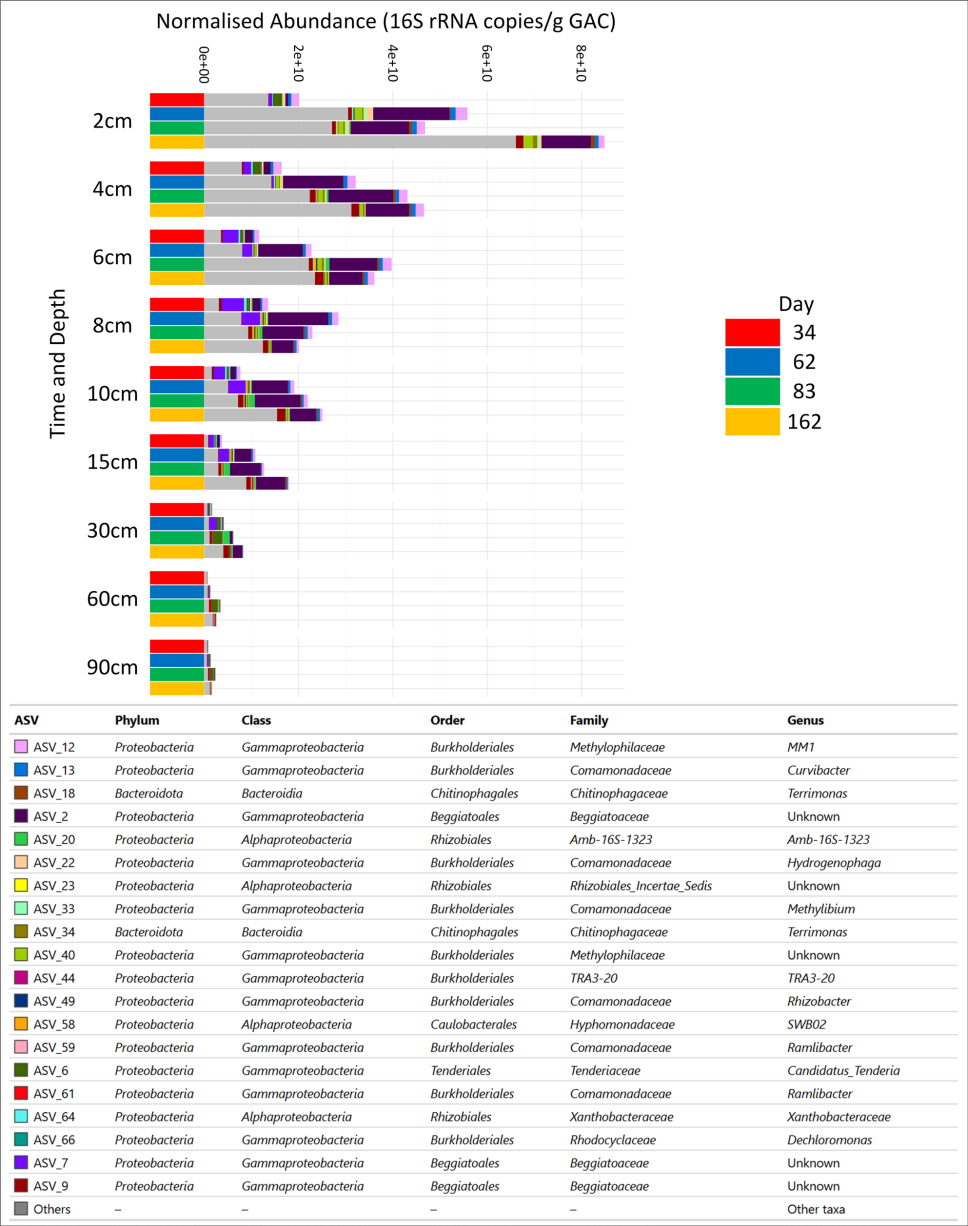
The 20 most abundant taxa normalised against 16S rRNA qPCR data over time and through bed depth. Taxonomy to of each ASV reported to genus level

To identify the taxa driving changes in diversity we explored the 20 most relatively abundant taxa through the depth of the filter bed and over time (Fig. 4). To identify taxa which may be contributing to increasing biomass, absolute abundance measures were calculated by normalising with 16S rRNA qPCR data.

The 20 most abundant ASVs were defined as dominant taxa, corresponding to a mean relative abundance greater than or equal to 0.5% across all samples. ASVs with lower mean relative abundance were classified as rare taxa. The 20 top ASVs accounted for between 20 ± SD8% and 80 ± SD5% of the total taxa considering all timepoints and depths. The overall composition changed little over time and was established early on but did vary with depth. In the top section of the filter (0-15 cm) ASVs from family Beggiatoaceae (ASV_2, ASV_7, ASV_9) were in highest abundance ranging from ASV_2, 3 ± SD1% to 51 ± SD7%, ASV_7, 0–29 ± SD25%, ASV_9, 0–6 ± SD4% of the total over time. In the bottom section (30-90 cm), ASV_6, an uncultured Tenderiaceae was the most abundant ranging from 0 to 33 ± SD9%. Taxa outside the 20 most abundant ranged from 20 ± SD5% to 80 ± SD8% of the total considering all depths and time points. These taxa were found to be rarer than the dominant 20 ASVs, decreasing to a range of 10 ± SD2% to 62 ± SD9% when the top 100 most abundant ASVs were identified. Indeed the 21st most abundant ASV, an unknown Rhizobacter ASV_49 averaged at an abundance of 0.8 ± SD0.5% when present and maximum abundance of 2 ± SD0.3%.

When transformed to absolute abundance the stratification throughout the filter bed becomes clear with the most abundant taxa in the top section (0–15 cm) accounting for 4.21 × 10^10^ ± SD1.09 × 10^9^ to 1.0 × 10 ± SD1.2 × 10^9^ copies/g GAC and between 24 ± SD9% and 77 ± SD7% of the total community; while in the bottom (30-90 cm) the top 20 taxa were a log fold lower at 1.33 × 10^9^ ± SD5.6 × 10^7^ to 8.93 × 10^9^ ± SD 2.27 × 10^8^ copies/g GAC, but still between 20 ± SD5% and 80 ± SD8% of the total community. Rare taxa, identified as others (Fig. 4), were also most abundant in the top section of the filter bed ending at a total 1.58 × 10^11^ ± SD1.91 × 10^7^ copies/g GAC by day 162 compared to only 7.05 × 10^9^ ± SD3.92 × 10^6^ copies/g GAC in the bottom sections (30-60 cm).

Over time the largest increase in most abundant taxa was observed between days 34 and 62 when they increased 140% (4.35 × 10^10^ ± SD1.09 × 10^9^ to 1.04 × 10^11^ ± SD9.97 × 10^8^ copies/g GAC.) This aligns with the period of highest growth as identified by mass balance calculations (Fig. 2). ASV_2 (uncultured *Beggiatoaceae*) was found to have increased the most over this timeframe in terms of absolute numbers. Increasing from 6.98 × 10^9^ ± SD9.25 × 10^8^ to 6.24 × 10^10^ ± SD2.48X10^9^, a 794% increase. While ASV_2 was highest in number, three other ASVs had higher percentage increases during the growth phase. ASV_18 (genus *Terrimonas*) increased 5603%, ASV_35 (uncultured *Rhizobiales*) increased 4259% and ASV_47 (uncultured *Gracilibacteria*) increased by 2568%. Two ASVs, ASV_40 (uncultured *Methylophilaceae*) and ASV_61 (uncultured *Ramlibacter*) were absent on day 34 but had increased to 6.63 × 10^7^ ± SD1.33 × 10^7^ (ASV_40) and 1.18 × 10^9^ ± SD2.15 × 10^7^ (ASV_61) copies/g GAC by day 62.

During the growth phase (day 34–62), rare taxa increased by 117% (3.27 × 10^10^ ± SD2.76 × 10^6^ to 7.11 × 10^10^ ± SD6.13 × 10^6^ copies/g GAC). Following this, from day 62 to 162 the rare taxa in the filter bed continued to increase by 131% to 1.65 × 10^11^ ± SD1.57 × 10^7^ copies/g GAC. Conversely, the most abundant taxa decreased by 25% over this period to 7.79 × 10^10^ ± SD4.75 × 10^8^ copies/g GAC by day 162.

**Fig. 5:**
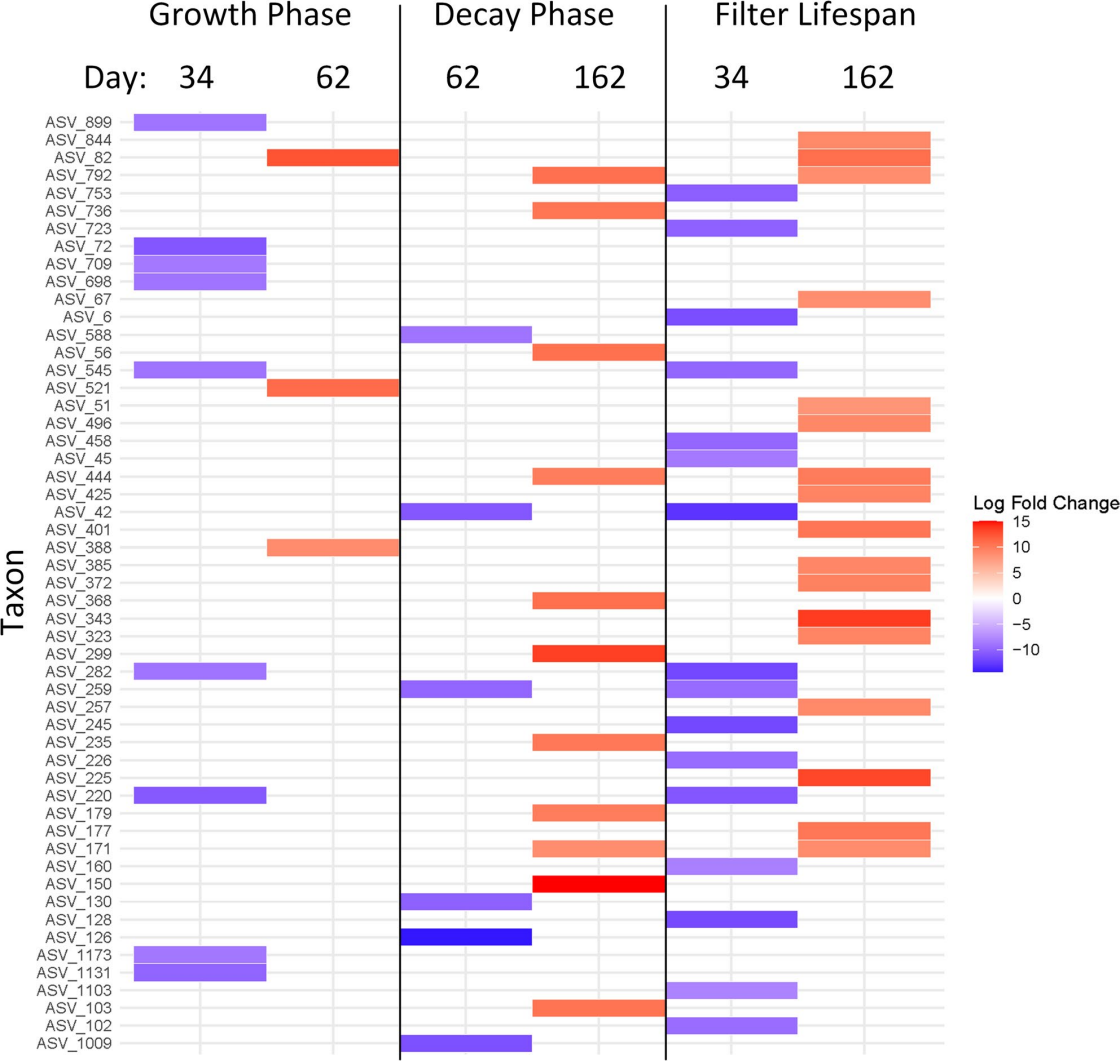
ANCOM-BC differential abundance analysis of ASVs with significant (Holm adjusted *p* value < 0.05) log fold changes (> 2). Columns offer comparisons between the different phases of biomass accumulation (Fig. 2A). The left column indicates taxa with significant log fold changes (> 2) during the growth phase (days 34–62). The middle column indicates taxa with significant log fold changes (> 2) during the decay phase (days 62–162). The right column indicates taxa with significant log fold changes (> 2) over the filter lifespan (days 34–162). ASV taxonomy can be found in Table S1

This suggests that following the initial growth phase (days 34–62, Fig. 2) the most abundant taxa in the filter bed generally cease increasing in absolute number, though persisted at high abundance through the later periods of overall decay (days 62–162, Fig. 2). However, total biomass in the filter bed continues to increase beyond the initial growth phase. This begs the question as to what taxa were driving these increases, particularly during these later phases of overall decay. To answer this question, a differential abundance analysis on the absolute abundance normalised data set was performed to identify taxa with significant log fold changes across the growth phase, decay phase and entire filter lifespan (Fig. 5).

Differential abundance analysis found 62 ASVs which had significant (Holm adjusted *p* value < 0.05) log fold changes across one or more of the three temporal phases investigated. Interestingly, the growth phase had fewest ASVs with significant log fold increases (lfc) (ASV_82 (unknown *Rhodospirillales*), lfc - 12.5 ± SE2.2, ASV_521 (unknown *Micavibrionales*), lfc - 11 ± SE2.1, ASV_388 (genus *Babeliales*), lfc - 8.8 ± SE2.2) despite this phase having the steepest increases in biomass. Indeed, this phase contained nine ASVs that significantly decreased in number between days 34 and 62.

During the later decay phase between days 62 and 162, 11 ASVs had significant log fold increases. Fewer ASVs in the decay phase were found to have decreased between days 62 and 162, despite overall cell loss during this timeframe. During both the growth and decay phases none of the 20 most abundant ASVs were found to have significant log fold changes.

Over the life span of the biofilter, the number of ASVs with significant positive and negative log fold changes were nearly in equilibrium, with 17 positive log fold changes to 16 negative. ASV_6, (genus *Candidatus_Tenderia*) was the only ASV from the top 20, to have a significant log fold decrease (lfc -11.5 ± SE2.2 between day 34 and 162). Indeed, of the 62 ASVs identified as having significant log fold changes, only a further 13 were found among the top 100 most relatively abundant taxa (ASVs 82, 72, 67, 56, 51, 45, 42, 245, 177, 150, 128, 126, 103). This again suggests that during the later phases of filter bed community development rare taxa were largely driving increases in biomass.

These results indicate an abundant core of primary colonisers (top 20 taxa) were seeded in the first 34 days of operation and further established during the growth phase. These taxa persist in high abundance but generally remain stable in number once established. Outside of this abundant community, the simultaneous gain and loss of rare taxa appears to be driving changes in biomass and diversity (Fig. 3).

### Temporal changes in core community driven by deterministically selected rare taxa

#### Core microbiome in the filter bed over time

**Fig. 6:**
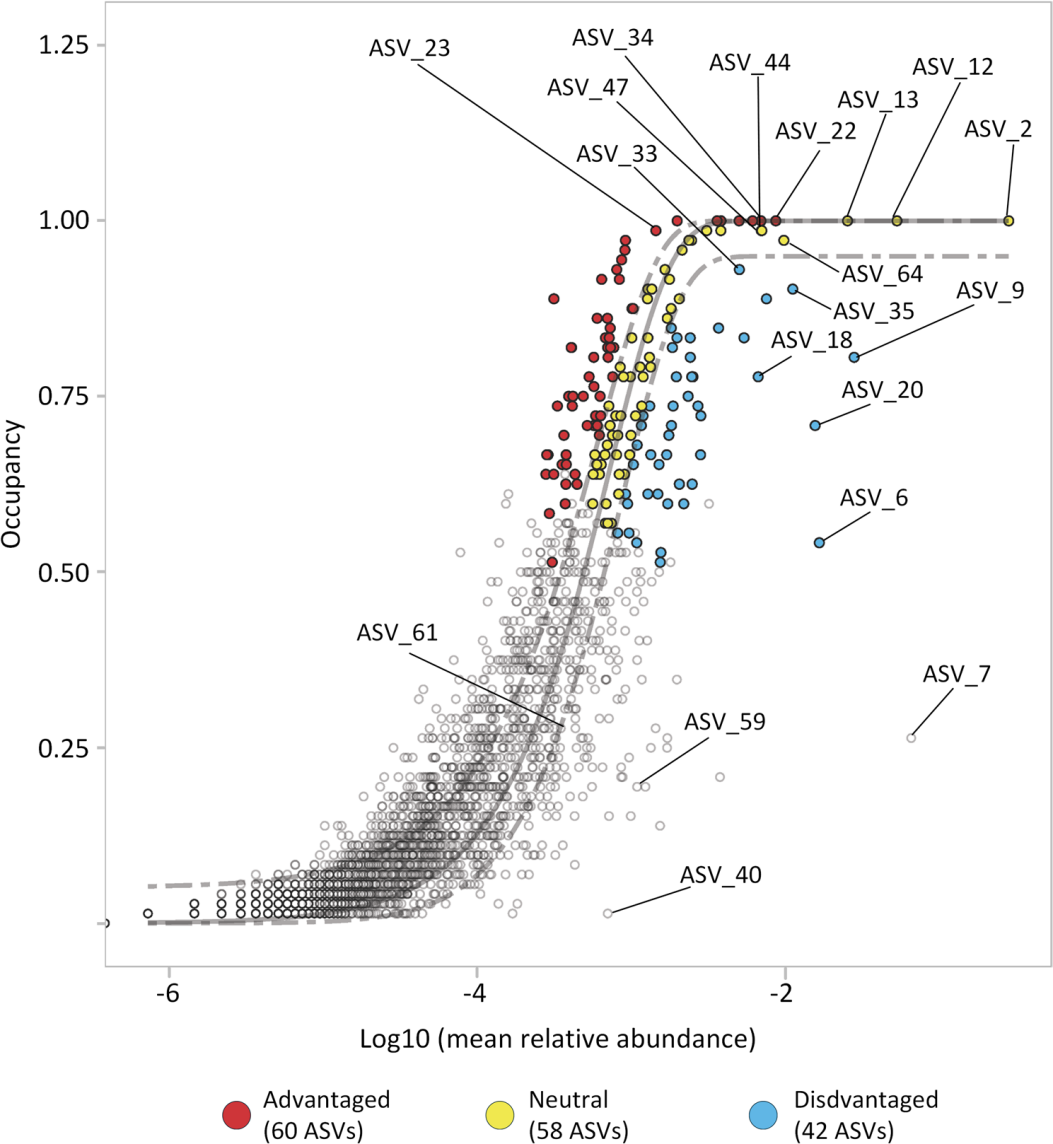
Sloan’s neutral community model (SNCM) showing the relationship between occupancy and mean relative abundance of the top section of the filter bed (15 cm) at all timepoints. The solid grey line represents the model-predicted frequency of occurrence for each taxon based on its mean relative abundance. Dashed lines indicate the 95% confidence intervals of the model. Coloured points denote the 160 ASVs belonging to the core microbiome. Open grey points denote non-core taxa. Points in red represent taxa occurring above the upper 95% prediction limit (more frequent than expected under neutral dynamics, termed here advantaged). Blue points lie below the lower 95% limit (less frequent than expected, termed here disadvantaged). Yellow points fall within the 95% prediction interval (taxa whose distributions are consistent with neutral expectations, termed here neutral). Named ASVs are among the 20 most abundant taxa across all timepoints and depths. Full taxonomy of ASVs within the core microbiome can be found in Table S2

We utilised core community analysis accompanied by neutral modelling to further test this hypothesis and identify if there was a core community within the filter bed, how this related to abundant and rare taxa and to identify how this core might be assembled. As the majority of biomass was found in the top section of the filter bed (0–15 cm), this section was the focus of analysis. A total of 160 ASVs were identified as the core microbiome of the top section (0–15 cm) of the filter bed across all timepoints (Fig. 6.) Of these, 58 were stochastically assembled and 102 assembled deterministically.

Slightly more of the deterministically assembled ASVs (Fig. 6) were advantaged by the filter environment (60) than disadvantaged (42). The core microbiome included 15 of the most abundant ASVs (ASVs 12, 13, 18, 2, 20, 22, 23, 33, 34, 35, 44, 47, 6, 64, 9) (see Fig. 5 for taxonomy). Of these, six, including the most dominant ASV2, were neutrally assembled and nine deterministically, with three being advantaged (ASVs 22, 23, 34) and the remaining six disadvantaged. Despite their persistence and abundance through all sampling timepoints only 20% of the most abundant ASVs present in the core microbiome were advantaged by the filter environment. Comparatively, 38% of rare taxa were advantaged by the filter environment across all timepoints.

#### Initial colonisation of filter bed from the water

The persistence of the most abundant taxa from day 34 raises the question of their origin. For this we examined the microbial community of the influent water seeding the biofilter over its first 34 days of operation (Fig. S4). PERMANOVA of weighted UniFrac found the influent water was statistically similar over the initial 34 days (*p* value = 0.084). As such we combined the glass fibre and 0.22 µm size fractionated communities collected weekly over the first 34 days of operation to derive a water core microbiome, termed the ‘seeding community’.

The core microbiome of the seeding community (383 ASVs) was compared to the core microbiome of the entire filter bed on day 34 (302 ASVs) revealing 119 shared ASVs. Of the shared ASVs only seven (ASVs 12, 2, 22, 23, 33, 34, 35) were among the 20 most abundant ASVs which persisted within the filter bed over time (Fig. 4). All seven were stochastically assembled in the filter bed by day 34. Conversely, four of these ASVs (ASVs 12, 2, 22 and 33) were deterministically assembled in the seeding community (Fig. S5). Of note, ASV_2, the most dominant organism in the filter bed over the filter run was advantaged in the influent water but neutrally assembled within the filter bed.

Of the 302 ASVs in the core community of the filter bed on day 34, 16 of the top 20 most abundant ASVs were present (12, 13, 2, 20, 22, 23, 33, 34, 35, 44, 47, 59, 6, 64, 7, 9) (Fig. 4). Of these, 11 were assembled stochastically (ASVs 12, 13, 2, 22, 23, 33, 34, 35, 44, 47, 6) while five were disadvantaged by the filter environment (ASVs 59, 64, 7, 9). None of the dominant and persistent taxa were advantaged by the filter environment.

Comparatively, of the remaining 286 rare ASVs, 31.12% (89 ASVs) were advantaged; 18.88% (54 ASVs) were disadvantaged and the remaining 50% of taxa were assembled stochastically. This suggests that abundant early colonisers are largely neutrally assembled while rare taxa may be more influenced by deterministic factors within the filter bed and more likely to be advantaged by the filter environment.

## Discussion

In this study, quantitative microbial biomass and community structure were monitored over the first 6 months of biofilter operation, alongside the microbial community of the influent water. The findings demonstrate that biofilter microbial assembly is a dynamic process characterised by alternating phases of growth and decay. The most abundant microorganisms originated early from the influent water through stochastic colonisation and persisted with relatively stable absolute abundances. In contrast, variation in total biomass, abundance, and diversity was primarily driven by the turnover of rare taxa, shaped by deterministic selection following their introduction from the influent.

### Microbial biomass accumulation

Microbial biomass within the filter bed exhibited distinct phases of growth and decline during the 6-month start-up period. Although total biomass increased overall, this reflected successive cycles of accumulation and loss rather than continuous growth. Early increases in biomass occurred without substantial changes in species richness, whereas later phases of biomass decline coincided with greater diversity, suggesting turnover among rare taxa influenced by evolving environmental conditions within the filter bed. Previous studies have attributed biomass fluctuations mainly to operational disturbances such as backwashing [18, 45]. In contrast, the present study demonstrates that, even in the absence of such interventions, start-up in slow flow biofilters is inherently dynamic, driven by the persistence of early colonisers and the deterministic selection of rare, transient taxa. An initial biomass growth phase was observed between days 34 and 62, during which biomass increased at its highest rate. This likely reflects a combination of favourable environmental conditions and microbial utilisation of nutrients [1, 42]. The subsequent decay phases (days 62–162) indicate that factors such as nutrient limitation due to biomass accumulation, competition for space, and community succession may play a role in regulating biomass levels [11, 19]. Continued increases in biomass and diversity through this later decay phase imply that the composition of the microbial community is in flux.

### Role of abundant and rare taxa in biomass dynamics

A key finding of this study is that the most abundant taxa do not appear to be driving later biomass increases following early establishment. The top 20 most abundant taxa accounted for a significant portion of the total microbial community (20 ± SD8% to 80 ± SD5%), yet their composition remained relatively stable over time, with limited changes in absolute abundance. This suggests that while these taxa were capable of persistence, they did not significantly contribute to the observed increases in biomass beyond the initial colonization phase. Instead, rare taxa exhibited significant fluctuations, with certain groups showing marked increases in absolute abundance during the later stages of the study. As demonstrated by differential abundance analysis, a greater number of ASVs exhibited significant log fold changes during the decay phase (days 62–162) compared to the earlier growth phase. These shifts imply that rare taxa play a crucial role in driving overall community changes and biomass accumulation as the bed community matures.

Previous studies have identified increases in diversity and changes in the microbial community with time [7, 16, 22, 23, 41]. However, these studies rely on relative abundance to detect temporal changes in the microbial community thus potentially masking important fluctuations in the absolute abundance of taxa. No previous study has utilised an absolute approach to interrogate the role of abundant and rare taxa in relation to temporal increases in biomass.

The persistence of abundant taxa despite their lack of significant increase in absolute abundance suggests that they form a stable core community. However, their role in later stage biomass dynamics appear limited compared to the rare taxa, which exhibit greater fluctuations in abundance. As a more diverse community has been linked to filter performance [7], these rare taxa may be of importance when aiming to optimise filter efficiency. It is important to acknowledge that this analysis relies on 16S rRNA DNA data, which cannot distinguish between active, dormant, or dead cells. As a result, an unknown fraction of the abundant taxa may reflect relic DNA from non-viable organisms. However, qPCR data of estimated taxa throughout the filter bed (Fig. S3) correlated strongly (Pearson's correlation coefficient 0.97, *p* value 1.0 × 10^–8^) with ATP data (Fig. 2). As such the observed increase in absolute numbers of rare taxa points to biological growth.

### Mechanisms of microbial assembly and selection

The core microbiome analysis provides further insights into how microbial communities establish and evolve within the filter bed. The presence of 160 ASVs in the core community though time, with a nearly equal mix of stochastically and deterministically assembled taxa, suggests a combination of neutral and selective processes in community formation throughout the whole lifespan of the filter [52]. However, during the early-colonisation phase (the initial 34 days), stochastic processes are more important: pioneer taxa drawn from the influent water establish themselves quickly on the freely available GAC surface mainly through stochastic mechanisms, most likely dispersal. This mirrors the virgin-GAC controls in Qin & Hammes, where the early community closely matched the influent and diverged only after adsorbed DOM created new niches analogous to what we observe after day 34 [33]. As our filter matured, deterministic selection became more prominent, especially among rare taxa. The heterogeneous DOM adsorbed onto the GAC suppling multiple carbon sources, enabling additional species to establish without displacing early dominants. This is confirmed by the resulting rise in the absolute abundance of rare taxa that also drove the observed increase in richness during later stages of maturation. Deterministic selection as a driver of community assembly, especially in established filters, has been reported in other studies. Lu et al. found that deterministic factors were responsible for the majority of community assembly at 60% compared to 40% stochastic assembly in full-scale BAC filters [21]. Li et al. also demonstrated that deterministic and stochastic community assembly varied by seasonality in full-scale BAC filters, with assembly becoming largely deterministic during colder months and stochastic during milder temperatures [17]. Here we report that the most abundant community members are largely assembled stochastically from the influent water and generally less influenced by deterministic factors than rare taxa. This is also evidenced by their generally stable absolute numbers over time.

The higher proportion of rarer taxa that were advantaged by the filter environment compared to the abundant taxa suggests that environmental selection pressures can favour less dominant community members over time. This may arise from factors such as substrate specialisation, metabolic flexibility, or resilience to environmental fluctuations [26, 36]. Similar observations, where rare taxa respond more strongly than dominant members to deterministic cues such as disturbance, have been reported in lakes, soils, and drinking-water distribution systems [6, 37, 39, 59].

The patterns observed in this study, where abundant taxa assembled largely through stochastic colonisation from the influent while rare taxa were shaped more strongly by deterministic selection, reflect ecological mechanisms common across engineered microbial systems. Although Wang et al. found the opposite trend in biofilters, with rare taxa more influenced by stochastic processes and abundant taxa by deterministic ones, both studies emphasise the ecological significance of rare taxa [54]. In the present work, rare taxa responded to selective pressures within the maturing filter and were key drivers of biomass accumulation and community diversification. Comparable trends have been observed in full-scale drinking-water BAC filters, where flow configuration and contact time modulate the balance between stochastic and deterministic forces and ultimately shape community function and pollutant removal [9, 53]. In industrial wastewater treatment systems, rare taxa also assemble deterministically and underpin network stability and functional resilience, while abundant taxa are more stochastically structured and less sensitive to environmental selection [55]. These parallels suggest that the partitioning of assembly processes between dominant and rare community members may represent a general property of engineered microbiomes. Evidence from activated-sludge and bioreactor systems further indicates that operational factors such as hydraulic regime, substrate loading, and solids retention time can shift this balance and thereby influence community function and system stability [44]. Collectively, the pattern observed here, involving stochastic assembly of abundant taxa and deterministic selection of rare taxa, fits within a broader ecological framework for engineered microbial communities. Understanding and managing these rare populations may therefore provide a unifying basis for improving microbial performance.

### Ecological implications and future considerations

These findings have clear engineering implications, but they also expose a cautionary point about how we assess “who matters” in a biofilter and emphasises the need to consider absolute and not just relative abundance changes. Our data show that late-stage biomass gains stem mainly from the active growth of formerly rare taxa, underscoring the importance of tracking less-dominant members, not just the most abundant ones present in the community. Additionally, we detected a dominant community whose absolute abundance remained remarkably constant from the start of filter operation. The persistence of this community may allow for the seeding of a purposefully engineered community. If a highly optimised and efficient community could be engineered and propagated within the filter bed, this work suggests it may be able to persist over time, resisting deterministic factors which appear to influence rare taxa.

That prospect, however, rests on a critical assumption. Because our evidence is based on amplicon DNA, some proportion of this “stable” signal could represent relic DNA from inactive or even dead cells. If so, the apparent resilience of this abundant community might overstate its functional relevance in the system. Future work should combine DNA profiling with activity-based measures to separate living, active contributors from inert genetic debris.

By contrast, the rare taxa clearly showed active growth (suggested by the measured changes in their absolute numbers), implying they responded to deterministic cues that emerged only in the filter’s later stages. If such deterministic factors within the filter bed could be better quantified and understood, it may be possible to utilise an engineered bed to target the later selection of useful rare taxa which may otherwise be difficult to seed early in the filter lifespan.

In conclusion, this study provides a comprehensive view of microbial community dynamics within a biofilter, revealing a complex interplay between growth, decay, and community succession. The findings emphasise the critical role of rare taxa in driving biomass increases and suggest that microbial communities within engineered systems are shaped by both neutral and selective forces. Understanding these processes can inform the optimisation of biofilter design and operation, ultimately enhancing microbial efficiency in water treatment and other biotechnological applications.

## Supplementary Information

Below is the link to the electronic supplementary material.

Supplementary Material 1

Supplementary Material 2

## Data Availability

Raw sequence files supporting the results of this article are available in the National Center for Biotechnology sequence read archive under project accession PRJNA1309034.

## Abbreviations

ASV: Amplicon sequence variant
ATP: Adenosine triphosphate
BAC: Biological activated carbon
DOC: Dissolved organic carbon
GAC: Granular activated carbon
