# Supplementary material for: Deterministically selected rare taxa drive changes in community composition in drinking water biological activated carbon filters
Source: Environ Microbiome. 2025 Nov 24;20:162. doi: 10.1186/s40793-025-00820-4 (PMC12751416; doi:10.1186/s40793-025-00820-4)
Supplement: Supplementary file 1 — Supplementary Material 1 [file 40793_2025_820_MOESM1_ESM.docx]

Deterministically selected rare taxa drive changes in community composition in drinking water biological activated carbon Filters

Dominic Quinn^a^, Marta Vignola^a^ Jeanine Lenselink^a^, Graeme Moore^b^, Stephanie Connelly^a^, Caroline Gauchotte-Lindsay^a^, Umer Ijaz^a^, William Sloan^a^, Cindy J. Smith^a^

*a*University of Glasgow – James Watt School of Engineering, G12 8QQ, United Kingdom*

*b* Scottish Water, 6 Castle Drive, Dunfermline, KY11 8GG, United Kingdom*

*Supplementary materials and methods*

*S1 Biofilter construction*

Each individual filter column was constructed from PE80 pipe and MDPE fittings. The main column length was cut from 32mm PE80 pipe. The length cut was the specified filter bed length (30cm, 60cm or 90cm) plus an additional 370mm. A 32mm MDPE pipe insert was wrapped in stainless steel mesh (aperture 75µm) and inserted to the end of the pipe. This end was fit to a 32mm x 25mm x 32mm MDPE reducing T. The other 32mm end of the reducing T was occupied by a 180mm length of PE80 pipe and 32mm MDPE terminal cap. The 25mm are of the reducing T was occupied by a 160mm length of PE80 pipe and 15mm to 25mm MDPE reducing stop cock. A 200mm length of 15mm BRAND® laboratory tubing and 15mm MDPE pipe insert was added to the stop cock. A 6mm-15mm reducer connected the 15mm tubing to 6mm hydrolysis resistant tubing and a 2mm – 5mm reducer connected this to 2mm PVC tubing. This tubing was connected to a Watson Marlow 300 series peristaltic pump with a 5 channel microcassette pump head. The filter columns were attached upright to a plywood frame with 32mm pipe clips. An 8mm hole was drilled 50mm down from the top of the filter column and 8mm hydrolysis resistant tubing was added as an overflow. 260g dry weight Cabot Norrit GAC 1240 W was added to a 1L glass bottle. The GAC was rinsed with 260ml Milli-Q water until the water became clear. Once clear the GAC and 260ml of Milli-Q water were sonicated for 10 minutes in an ultrasonic bath. The GAC was left to settle in water for 48 hours. The GAC was then added to the filter columns in small batches with Milli-Q water and pushed down until tightly packed. This continued until the filter bed was the desired length. The biofilters were then allowed to cycle with Milli-Q water for 2 weeks to ensure the GAC was fully settled. Influent water was collected from a Scottish Water surface reservoir, filtered on site through 10µm using a submersible pump. Approximately 500L was collected every 2 weeks and stored at room temperature. Influent was fed into the filter from a 20L jerrycan using a Watson Marlow 300 series peristaltic pump and 6mm hydrolysis resistant tubing until flowing freely from the overflow tubing. A single jerrycan of influent fed each set of triplicate reactors and was replaced at the start of each week. The peristaltic pump at the effluent end of the filter was set at a consistent flow rate of 1ml/min across all biofilters.


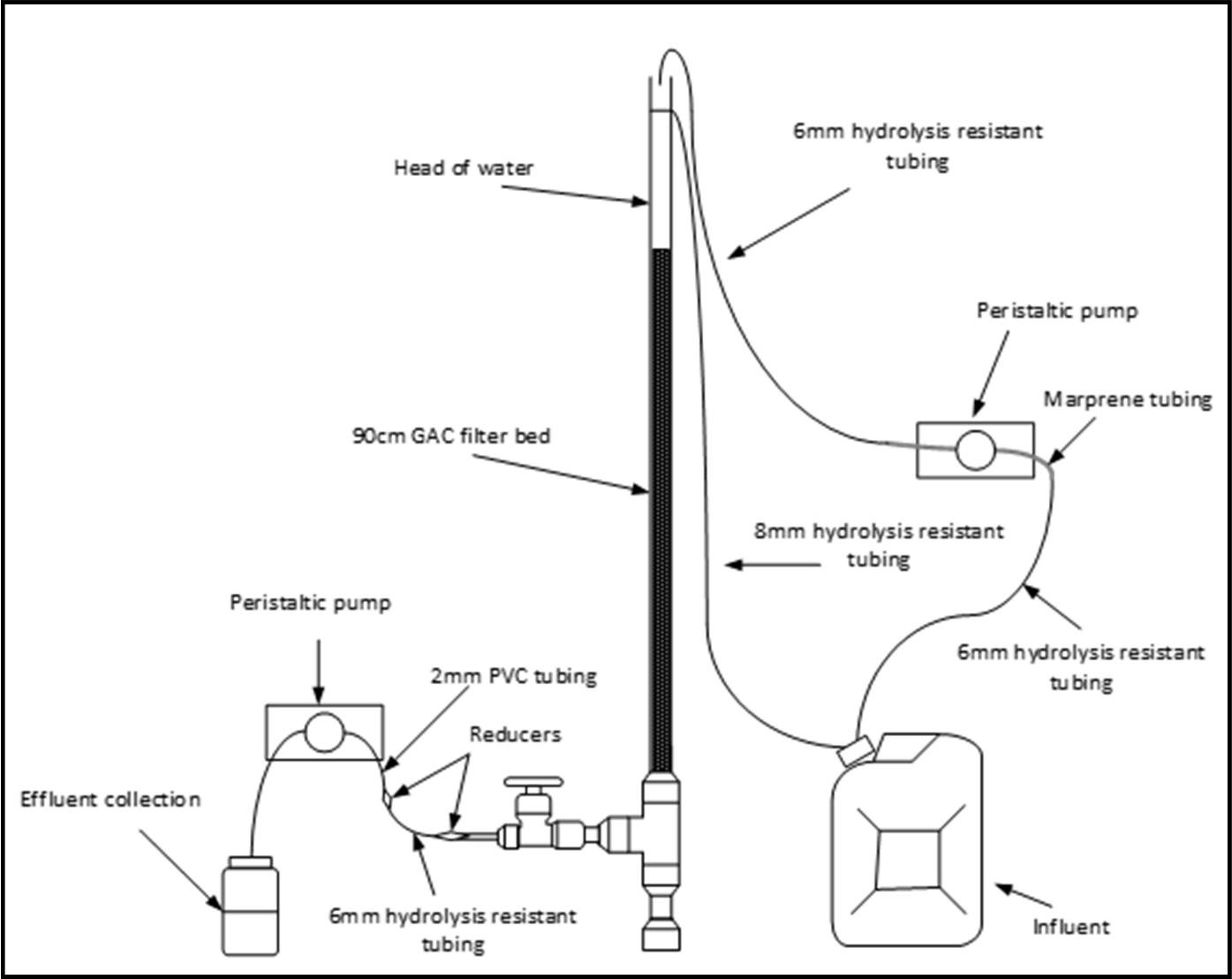


**Figure S1.** Diagram of biofilters operated in this study with 90cm GAC bed.

*S2 Biofilter deconstruction, sampling and water filtration*

Triplicate biofilters were taken down, drained and deconstructed through the filter depth (0-2cm, 2-4cm, 4-6cm, 6-8cm, 8-10cm, 13-15cm, 28-30cm, 58-60cm and 88-90cm) after 34, 62, 83 and 162 days of operation (Figure 1) using 32mm pipe cutters sterilised with 70% ethanol. GAC from each 2cm section was collected in sterile 200ml Corning tubes and homogenised. For immediate ATP analysis (BacTiter-Glo™, Promega, Madison, WI, USA), 400 mg was used, while the remaining sample was aseptically aliquoted into 2 ml cryotubes (0.5 g GAC per tube) and stored at −80 °C until further processing.

From the start of the experiment until the first deconstruction at day 34, two litres of influent water were collected weekly and size-fractionated by inline filtration. The water was first passed through a 1.2µm MF200 glass microfibre filter ( Fisher brand, Loughborough, UK) held in a 47mm Swinnex filter holder (Merck Millipore, Darmstadt, Germany), followed by filtration through a 0.22 µm Sterivex™ filter (Merck Millipore, Darmstadt, Germany). All tubing and filter holders were sterilised by autoclaving for 15 minutes at 120ºC before filtration. Both filter fractions were stored at -80ºC until DNA extraction.

*S3 Flow cytometry total and intact cell counts – influent and effluent water*

Weekly, 3ml of influent and effluent water was sampled in a sterile 15ml centrifuge tube. The sample was fixed with 3ml of 1% v/v glutaraldehyde in ultrapure MilliQ water and stored in the dark at 4°C and analysed within 48 hours. For total cell count, 1ml of fixed sample was stained with 10µl of SYBR Green I ® (Invitrogen, Thermo Fisher Scientific, Waltham, MA, USA)10,000 x in ≥99.9% DMSO (Merck KGaA, Darmstadt, Germany) (1:100 dilution in Tris-EDTA buffer solution, pH 8.0). For intact cell counts, 1ml of fixed sample was stained with 10µl of SYBR Green I® and propidium iodide solution (Invitrogen, Thermo Fisher Scientific, Waltham, MA, USA) (1:100 dilution of SYBR Green; 0.6 mM Propidium Iodide in 0.22µm filtered Tris-EDTA buffer solution). Stained samples were vortexed for 5 seconds and incubated in the dark for 13 minutes at 37°C. Samples were analysed on a BD Accury™ C6 Plus flow cytometer (BD Biosciences, Franklin Lakes, New Jersey, USA) equipped with a 488nm laser; the flow rate was fixed at 100µl/min, analysing 50µl of the sample. Following analysis, the data were extracted in the Flow Cytometry Standard format and imported into R (version 4.3.1) using the FlowCore package. The strategy for distinguishing stained cells from both the instrument's background and the water's organic signals involved virtual gating on the FL1(533/30 nm) and FL3 (670 nm) fluorescence channels. To design the electronic gate, freshly filtered (0.22µm) samples of ultrapure Milli-Q water, of 1% v/v glutaraldehyde solution and of influent and effluent water from each sampling timepoint were utilised.

**Figure S2.** Visual representation of sampling for the mass balance analysis. Total cell counts by flow cytometry of the influent and effluent water were measured weekly between each deconstruction timepoint. Cells estimated by ATP analysis were analysed from GAC collected at each deconstruction timepoint. For sections of the filter bed which were not sampled (e.g. 30-58cm) cell numbers were extrapolated upwards from the lower section of the filter bed (e.g. 58-60cm).

*Supplementary Results*


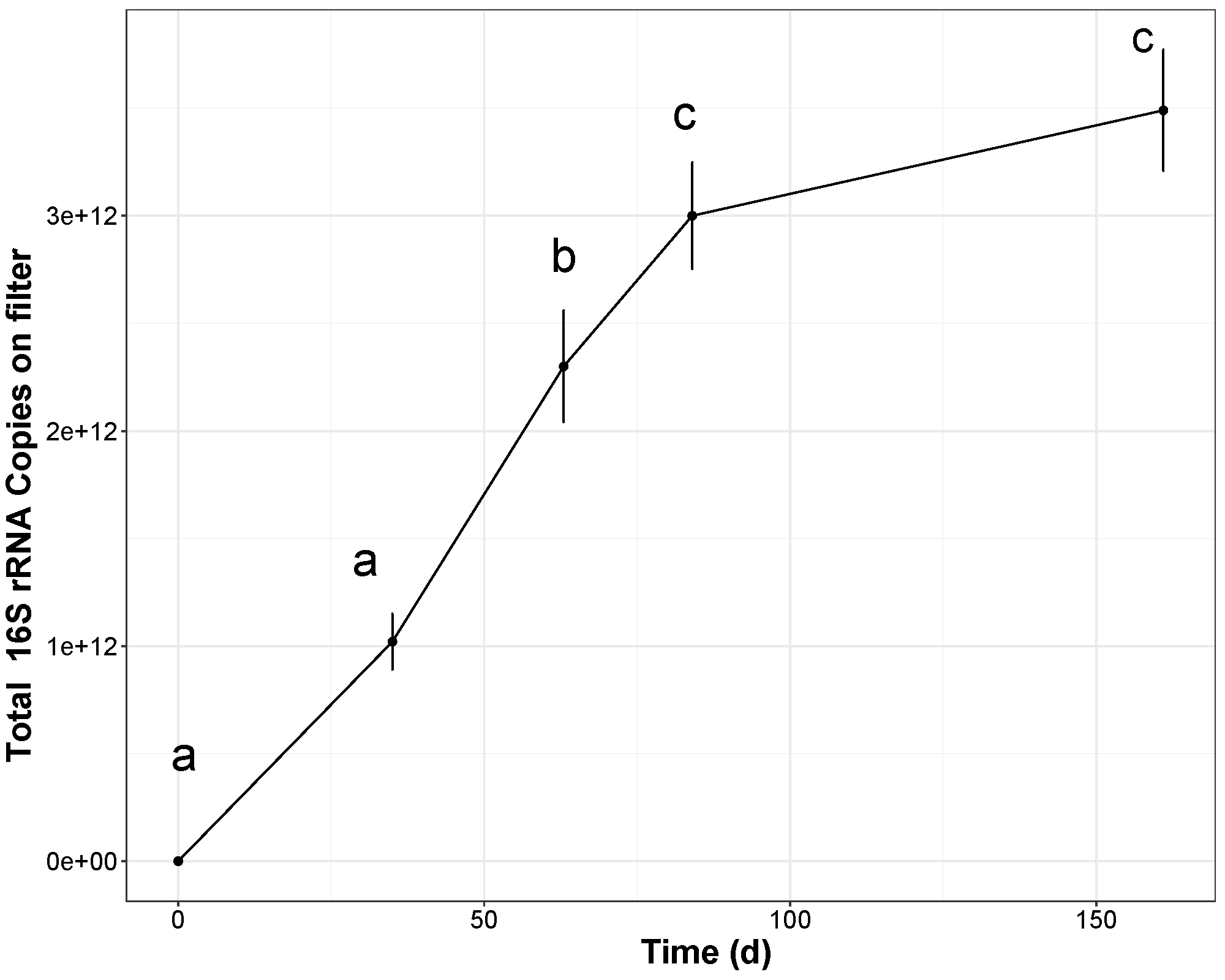


**Figure S3.** Biomass found on the entirety of the filter column over time measured by QPCR 16S rRNA gene copy numbers per filter. Error bars show standard deviation and letters of significance were generated by one way ANOVA and Tukey HSD.


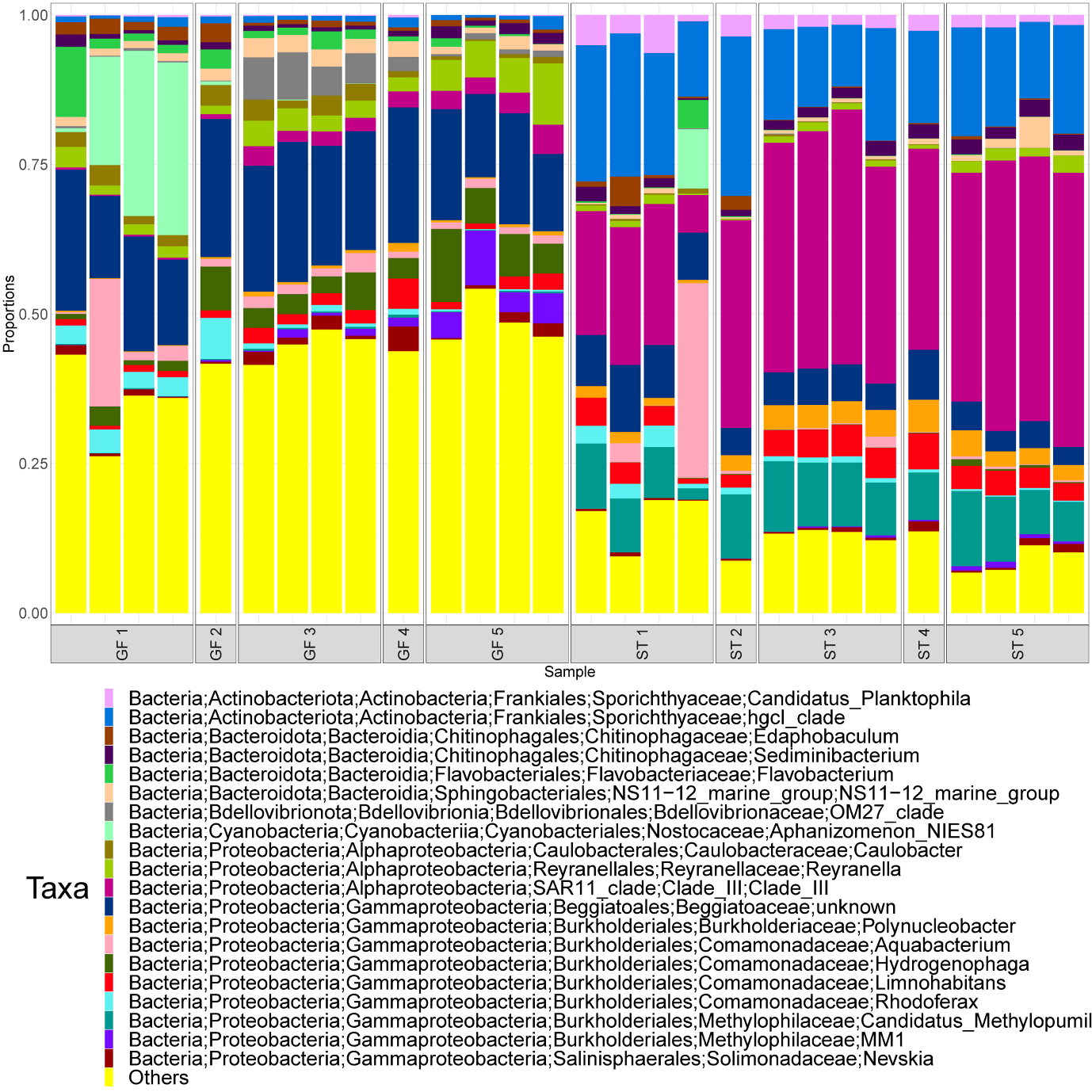


**Figure S4.** The 20 most relatively abundant taxa at Genus level of the seeding community by sampling week (1, 2, 3, 4, 5) and size fractionation (GF – glass fibre 1.2µm retention, ST – Sterivex filter 0.22µm retention)

**
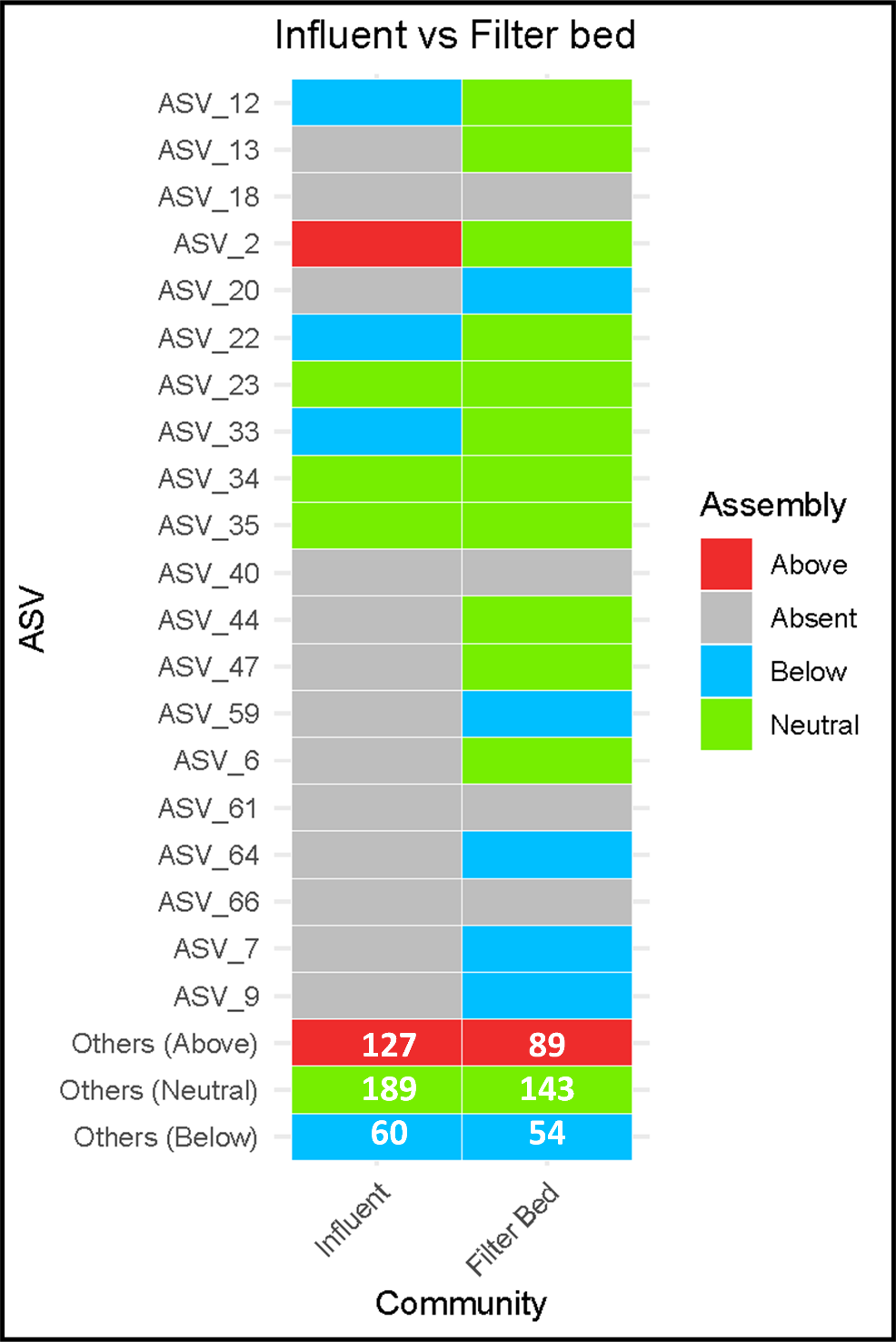
**

**Figure S5**. Comparison of the core microbiome of the filter bed at week 34 and the seeding community (influent water sampled weekly till day 34). Values in “Other” taxa boxes reflect the number of ASVs in the core microbiome assigned to each mode of assembly. Taxonomy for ASVs within each core microbiome can be found in Tables S3 and S4.
